# Slow Proton Transfer Coupled to Unfolding Explains the Puzzling Results of Single-Molecule Experiments on BBL, a Paradigmatic Downhill Folding Protein

**DOI:** 10.1371/journal.pone.0078044

**Published:** 2013-10-28

**Authors:** Michele Cerminara, Luis A. Campos, Ravishankar Ramanathan, Victor Muñoz

**Affiliations:** 1 Centro Nacional de Biotecnología, Consejo Superior de Investigaciones Científicas, Madrid, Spain; 2 IMDEA-Nanociencia, Madrid, Spain; 3 Department of Chemistry and Biochemistry, University of Maryland. College Park, Maryland, United States of America; University of Leeds, United Kingdom

## Abstract

A battery of thermodynamic, kinetic, and structural approaches has indicated that the small α-helical protein BBL folds-unfolds via the one-state downhill scenario. Yet, single-molecule fluorescence spectroscopy offers a more conflicting view. Single-molecule experiments at pH 6 show a unique half-unfolded conformational ensemble at mid denaturation, whereas other experiments performed at higher pH show a bimodal distribution, as expected for two-state folding. Here we use thermodynamic and laser T-jump kinetic experiments combined with theoretical modeling to investigate the pH dependence of BBL stability, folding kinetics and mechanism within the pH 6–11 range. We find that BBL unfolding is tightly coupled to the protonation of one of its residues with an apparent pK_a_ of ∼7. Therefore, in chemical denaturation experiments around neutral pH BBL unfolds gradually, and also converts in binary fashion to the protonated species. Moreover, under the single-molecule experimental conditions (denaturant midpoint and 279 K), we observe that proton transfer is much slower than the ∼15 microseconds folding-unfolding kinetics of BBL. The relaxation kinetics is distinctly biphasic, and the overall relaxation time (i.e. 0.2–0.5 ms) becomes controlled by the proton transfer step. We then show that a simple theoretical model of protein folding coupled to proton transfer explains quantitatively all these results as well as the two sets of single-molecule experiments, including their more puzzling features. Interestingly, this analysis suggests that BBL unfolds following a one-state downhill folding mechanism at all conditions. Accordingly, the source of the bimodal distributions observed during denaturation at pH 7–8 is the splitting of the unique conformational ensemble of BBL onto two slowly inter-converting protonation species. Both, the unprotonated and protonated species unfold gradually (one-state downhill), but they exhibit different degree of unfolding at any given condition because the native structure is less stable for the protonated form.

## Introduction

During decades, our view of protein folding was constrained by the lack of information on fundamental aspects that limited the interpretation of experimental data. A most important issue referred to the determination of how cooperative is protein folding, or in other words, how closely it adheres to a two-state process. Early kinetic experiments showed that many single-domain proteins fold in seconds to minutes [1]. Relative to bimolecular gas-phase reactions, such slow folding rates were taken as evidence that the unfolded and native states were separated by a high free energy barrier, in line with the two-state model [2]. The subsequent generalization of the two-state model for the analysis of protein folding thermodynamic [3] and kinetic [4] data led to an oversimplified view in which any deviations from two-state were ascribed to experimental uncertainty. It was then naturally assumed by experimentalists that folding of single domain proteins must be inherently cooperative [1]. In contrast, energy landscape theory proposed that the source of folding free energy barriers is a non-synchronous compensation between the decreases in conformational entropy and stabilization energy associated to folding reactions, and thus that these barriers are inherently small [5]. Theory also predicted that in some instances folding could proceed by diffusion on a barrier-less free energy landscape (downhill folding) [6]. These ideas coincided with the observations from early computer simulations using coarse-grained models, which systematically produced extremely low folding cooperativity [7]. As a consequence, a deep gap existed between experiment and theory in protein folding.

Such state of affairs has drastically changed over the last 15 years. Development of ultrafast kinetic methods allowed measurement of the timescales for secondary structure formation [8], [9], loop closure [10] and hydrophobic collapse [11], leading to estimates for the folding speed limit of about 1 µs [12] (i.e. about 7 orders of magnitude slower than molecular collisions in the gas phase). The same folding speed limit was independently obtained from the observation of a fast “molecular” phase in kinetic experiments of microsecond folding proteins [13]. Further independent confirmation for a ∼1 µs speed limit has come from the observation of strong size-scaling effects on the folding rates of single-domain proteins [14]. Converting these speed limits onto a pre-exponential term for the characteristic folding rate expression (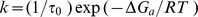) permitted the thermodynamic analysis of the activation free energy, so that contributions from conformational entropy, stabilization enthalpy and solvation free energy can be weeded out [15]. Such analysis applied to several slow-folding proteins confirmed empirically that folding barriers do arise from early loss in conformational entropy and also are rather small, as predicted by theory. By the same token, a ∼1 µs speed limit immediately leads to the classification of the many microsecond folding proteins identified in the last years as downhill or near-downhill folders [16].

In parallel, advances in analytical procedures based on statistical mechanics have rendered novel procedures for the interpretation of thermodynamic unfolding data. These procedures interpret certain deviations from two-state behavior in terms of populations of the conformational ensemble separating the native from the unfolded state (i.e. the thermodynamic free energy barrier to folding) [17]. For instance, the analysis of multiprobe equilibrium unfolding data with a statistical mechanics model provided the first experimental identification of downhill folding [18]. The assignment to downhill folding was later confirmed at full atomic resolution using nuclear magnetic resonance (NMR) [19]. Similar approaches applied to differential scanning calorimetry (DSC) experiments have shown that it is possible to distill these ideas onto analytical methods for estimating folding free energy surfaces (at least one-dimensional ones) and thermodynamic barriers [20]. Thermodynamic barriers so obtained correlate with the folding rates determined kinetically [21], supporting the direct link between fast kinetics and the equilibrium manifestations of marginal barriers and downhill folding [17].

Possibly of most practical interest is the global downhill folding limit, in which there is no significant free energy barrier (i.e. barriers below 1 *RT* ) at any experimental condition [22]. Under this scenario, the protein unfolds gradually by populating a single conformational ensemble that exhibits a degree of native structure that is proportional to the level of denaturing stress (one-state downhill folding) [23], [24]. At the denaturation midpoint, one-state folding proteins thus display a single ensemble of half-unfolded conformations rather than a 50–50 mix of native and unfolded molecules [23]. It is important to notice that, although intrinsically gradual, one-state unfolding still produces sigmoidal equilibrium unfolding curves [25]. The main difference is that the pre- and post-transition regions of the denaturation curve contain significant degrees of unfolding rather than being the phenomenological baselines predicted by the two-state model [26].

One-state downhill folding has been studied in depth on the small α-helical protein BBL using a variety of techniques and approaches [27]. For example, BBL exhibits broad sigmoidal unfolding with highly skewed pre- and post-transitions under both temperature and chemical denaturation [25]. The broad unfolding curves are also associated with dependence on the structural probe that is employed to look at unfolding [18], and an extremely broad distribution of atomic unfolding behaviors [19]. Similarly, the DSC thermogram for BBL is characteristically broad and with significant excess heat capacity at low temperatures, showcasing its gradual unfolding [20]. The microsecond folding kinetics of BBL have also been investigated using laser T-jump kinetic methods combined with multiple structural probes [28]. These kinetic experiments showed that downhill processes could produce single-exponential decays with similar rates for multiple structural probes rather than the stretched exponential decays expected for rugged downhill energy landscapes [6]. The one-state behavior of BBL was instead apparent in the kinetic amplitudes, which were very different for probes sensitive to secondary (IR) and tertiary (FRET) structure, indicating large decoupling between the two [28]. As yet another diagnostic manifestation of one-state downhill folding, it has recently been shown that the folding relaxation rate measured for BBL strongly depends on the magnitude of the perturbation [29].

The special features of one-state folding should be most apparent at the single-molecule level, where one can measure the distribution of molecular behaviors. For instance, single-molecule FRET spectroscopy, which recapitulates the conventional chemical denaturation bulk experiments for individual molecules [30], could in principle resolve whether a protein unfolds as a gradually shifting single state, or by the conversion between two end-states. There are, however, technical impediments since the typical timescales of downhill folding (microseconds) are too fast for even the most advanced SM-FRET methods, which can barely detect ∼1 photon per µs from a single molecule [31]. For BBL, this problem has been circumvented performing SM-FRET experiments at a temperature low enough to slow down its folding kinetics by nearly 100-fold. When carried out at pH 6, SM-FRET experiments unambiguously demonstrated the one-state scenario for BBL unfolding [32]. However, similarly low temperature SM-FRET experiments performed previously on BBL at higher pH have revealed a denaturant-induced conversion between two species with higher and lower FRET efficiency (*E*) [33].

The differences between the two sets of SM-FRET experiments are very puzzling, since they appear to be inconsistent with one another. As possible explanations for this discrepancy, it has been argued [34], and counter-argued [35], that the unimodal FRET efficiency (*E*) histograms observed at pH 6 could correspond to an experimentally unresolved mix of native and unfolded species. On the other, hand it has been noted that the experiments that produced a bimodal distribution exhibit several unconventional features [36]. For example, the two species detected in the latter experimental set exhibit marked decreases in *E* with denaturant [33], suggesting that each species undergoes partial unfolding. In addition, the denaturation midpoint obtained from the SM-FRET populations does not agree with the one determined by bulk experiments [36]. Another puzzling general result is the strong temperature dependence of the BBL folding kinetics, which seems to slow down by up to 200-fold with a ∼50 K temperature drop [32], [33].

Along these lines, it has been recently proposed that the answer to the BBL conundrum might be connected to an effective coupling between unfolding and proton transfer [35]. This idea has been put forward after the realization that BBL stability changes significantly in the pH 6–9 range [34], [35]. Such change in stability could be highly significant given that the effect of the ionic denaturant guanidinium chloride (GdmCl) on the *pK_a_* of buffers and on the pH reading of glass electrodes implies that the Huang et al. experiments were in fact performed at pH∼8 instead of 7 [35], compared to the pH 6 of Liu et al. Strong changes in stability between pH 6 and 9 are highly unusual since neither the acidic nor the basic residues of folded proteins titrate in that range. Moreover, in contrast to temperature and chemical denaturants, pH denaturation is an intrinsically binary process mediated by the conversion between the unprotonated and protonated forms of the protein. The binary character is most obvious when unfolding is induced by protonation of a single ionizable group with large *pK_a_* shift. Therefore, protonation could split an otherwise one-state ensemble onto two distinct gradually unfolding species [35]. Interestingly, a potential role for pH in determining the degree of BBL unfolding cooperativity was advocated previously on the basis of the differences in the NMR structures obtained at mildly acidic and neutral pH conditions [24].

Here we set out to investigate the role of proton transfer in BBL unfolding. Our main focus is twofold. First we aim to determine whether BBL unfolding is indeed coupled to specific protonation in the unusual pH 6–11 range; and second to study whether such effect could explain quantitatively both sets of single-molecule experiments as well as all previously available experimental data on BBL. For this endeavor we combine experiments and statistical mechanical modeling. We perform thermodynamic and laser T-jump kinetic experiments to elucidate the role of proton transfer in the chemical unfolding of BBL. We then explore the thermodynamic, kinetic and single-molecule implications of the coupling between unfolding and proton transfer on the one-state downhill folding scenario. For the latter we use the same one-dimensional free energy surface models that we have used before to: 1) analyze the thermodynamics and kinetics of protein folding [28], [37], [38], 2) interpret the deviations from two-state kinetics of fast-folding proteins [17], 3) analyze DSC experiments [39], and 4) predict folding and unfolding rates of single-domain proteins [40], [41]. Finally, we demonstrate how a straightforward mechanism of proton transfer coupled to unfolding nicely explains all of the existing experimental data on BBL within the context of the one-state downhill folding scenario.

## Results and Discussion

### The Stability of Native BBL Exhibits Unusual pH Dependence

The small protein BBL unfolds at mildly acidic pH due to the ionization of two buried histidines (H13 and H37) combined with the protonation of multiple acidic residues [42], [43]. Here we are interested in exploring possible destabilization effects in the neutral to basic pH range. First it is important to notice that at room temperature and between pH 6 and 11 the native state of BBL is stable according to the two-state analysis of thermal denaturation data [43]. BBL stability will be even higher at ∼279 K (our temperature of interest to compare with the previous SM-FRET experiments). To investigate the effects of pH in BBL stability at 279 K we thus investigated its resilience to an additional thermodynamic perturbation at various constant pH values within our range of interest. Particularly, we measured chemical denaturation curves using GdmCl as denaturant to better compare with SM-FRET experiments. Our choice of GdmCl over urea was imposed by the little sensitivity of BBL to urea [32] and the need to reach its complete unfolding under pH conditions of high intrinsic stability of the native state. However, due to the ionic character of GdmCl, the multimolar concentrations that are required to unfold BBL induce large changes in the pH of protein solutions prepared with the buffer conditions employed for standard protein denaturation experiments [44]. Moreover, evaluation of these pH changes using conventional methods requires careful correction for the effects of GdmCl on the readout from glass-electrodes [44]. To be as accurate as possible, we adjusted the pH for each sample of the denaturation curve individually using the reading of a glass-electrode corrected with the Garcia-Mira and Sanchez-Ruiz tabulation [44]. We monitored unfolding by far-UV CD, which is sensitive to the degree of native α-helix content present in BBL.

**Figure 1:**
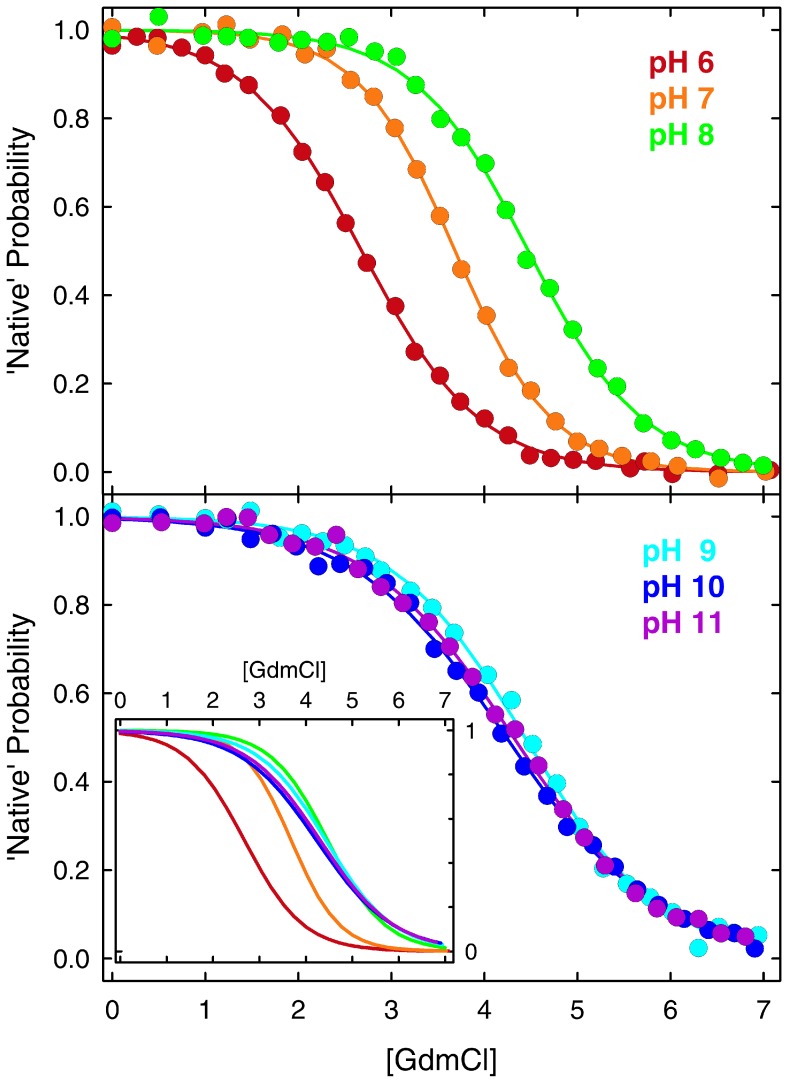
Equilibrium chemical denaturation of BBL at different pH values in the 6–11 range. The data is shown in terms of the probability of the native state obtained from fitting the experimental datapoints (circles) to a two-state model. The inset in the bottom panel shows the two-state fits for all the data together to facilitate comparison.

Fig. 1 shows the BBL native probability obtained from conventional two-state fits of the GdmCl denaturation curves at the various pH values. The fits indicate that, according to the two-state model, the BBL native state is stable in the absence of chemical denaturants across the entire 6–11 pH range. Nevertheless, the data show a strong stabilization of BBL in going from pH 6 to 8. The stabilization is manifested by increases in C_m_ (concentration of denaturant at which the protein is half unfolded), which goes from 2.7 to 4.5 M (Fig. 1, top panel). Above pH 8 the changes in BBL stability go in the opposite direction and are milder. In this second regime destabilization is manifested as lower m-values (the sensitivity to denaturant), which decrease from the pH 7 maximum of 4.5 kJ·mol^−1^·M^−1^ down to ∼2.9 kJ·mol^−1^·M^−1^ at the highest pH. In contrast, the C_m_ exhibits minimal changes in this pH range (Fig. 1, bottom panel). The overall trends are better observed in the inset of the bottom panel of Fig. 1, which overlays the curves obtained for the two-state fits for all the pH values rather than the experimental data.

The strong stabilization between pH 6 and 8 suggests the presence of an ionizable group with apparent *pK_a_* ∼7 that destabilizes the BBL native state upon protonation. The fact that the sensitivity to GdmCl denaturation is also maximal at pH 7 (m-value = 4.5 kJ·mol^−1^·M^−1^ at pH 7 versus ∼3.7 kJ·mol^−1^·M^−1^ at both pH6 and 8) further reinforces the idea that protonation of that residue does promote BBL unfolding. Such pH dependence is highly unusual for proteins, as most ionizable groups titrate at either lower or higher pH. We can eliminate the two buried histidines as the source for this behavior because they have lower apparent *pK_a_* according to determination by two independent groups [42], [45]. The various glutamates and aspartates present in BBL also display much lower *pK_a_* values [43]. By simple elimination, the titrating group (or groups) must then belong to a basic residue in BBL (lysine or arginine). Most of the positively charged residues in BBL are solvent exposed. However, calculation of the electrostatic potential of the BBL structures obtained by NMR at pH 7 [46] and pH 5.3 [19] show that the side-chain of R28 at pH 7 and R31 at pH 5.3 are involved in tertiary contacts and surrounded by a strongly positive potential which could be the structural source for a strong *pK_a_* downshift in the native state. The downshift on the fully native structure must be even larger since the apparent *pK_a_* ∼7 corresponds to the weighted average between the native *pK_a_* and that of unfolded BBL (presumably close to the *pK_a_*∼12 of an unperturbed arginine). In other words, simple physical reasoning indicates that the native state of BBL stabilizes the uncharged form of this residue. Protonation is thus very tightly coupled to BBL unfolding. The very mild destabilization at higher pH values could reflect the titration onset for some of the remaining basic residues in BBL, which could destabilize the BBL native state by non-specific electrostatic repulsions due to the positive net charge of the protein under these conditions.

**Figure 2:**
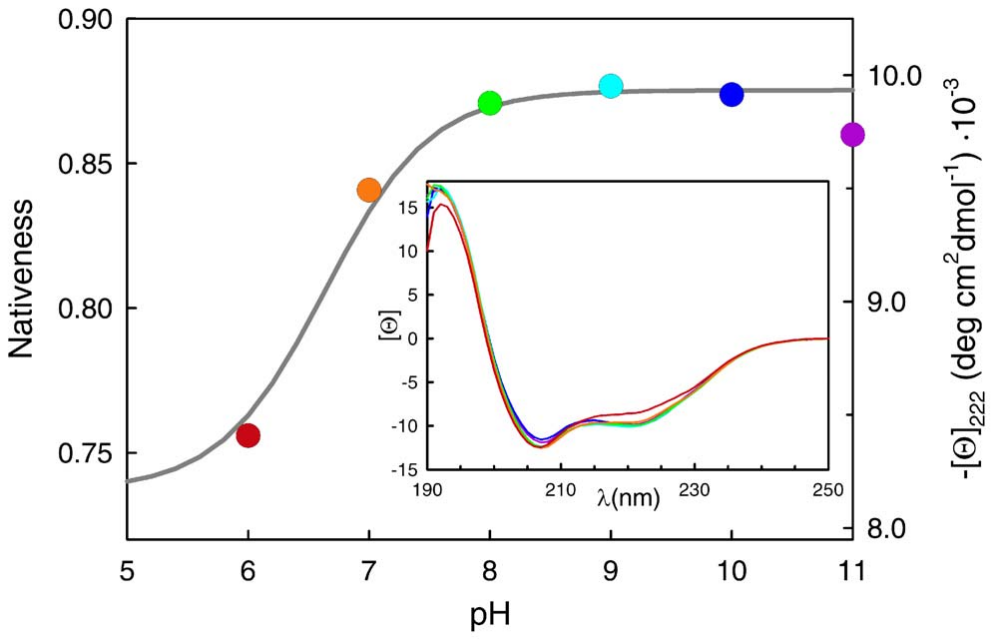
Changes in the native state of BBL as a function of pH. The right axis and the circles correspond to the mean residue ellipticity at 222-UV circular dichroism spectra of BBL at 279 K in which the color signifies the pH following the same code of the main figure and Fig. 1.

In addition to the unusual stability effects, pH seems to induce structural changes in the native state of BBL. This result is also somewhat peculiar, and seemingly incongruent with the two-state analysis. The data in Fig. 1 are shown in normalized fashion (i.e. native probability from two-state fits) to highlight that BBL reaches a fully native state at all pH values in the absence of chemical denaturant, as indicated by the thermodynamic two-state analysis (including pH 6, which has the lowest C_m_). However, direct inspection of the absolute far-UV CD signal for each condition reveals a sharp increase in the total α-helix signal of the native ensemble of BBL between pH 6 and 8, followed by an almost flat horizontal trend at higher pH (Fig. 2, circles, right scale). It is not possible to resolve the other end of the presumably sigmoidal titration because the two histidines, the glutamates and the aspartates start to titrate below pH 6, thus participating in the gradual unfolding of BBL [45]. It is also noteworthy that the changes in far-UV CD have clear spectral signatures that indicate that the thermodynamic native state of BBL (defined according to a two-state analysis) becomes gradually unstructured below pH 8 (inset to Fig. 2). Moreover, the curve shown in Fig. 2 is consistent with the apparent *pK_a_* ∼7 estimated from the changes in BBL stability, indicating that the native α-helix signal of BBL is tightly coupled to its stability. The pH effect on native BBL is thus similar to what has been reported before for temperature [22] and chemical denaturants [25]. This behavior coincides exactly with the general expectation for one-state downhill folding transitions, in which the degree of structure in the populated ensemble changes proportionally to the denaturing stress even in the pre-transition region [23]. Further evidence for gradual unstructuring of the BBL native state in this pH range is provided by comparison of the NMR structures at pH 5.3 [19], [47] and pH 7 [46].

### Kinetic Coupling between BBL Folding-Unfolding and Proton Transfer Processes

One of the intriguing characteristics observed for the folding-unfolding relaxation kinetics of BBL is its rather strong temperature dependence. The BBL folding-unfolding relaxation time at room temperature is about 20 µs [28]. At 279 K, however, it decreases down to 200 µs [32], or even 350 µs [33], depending on the actual pH of the sample under examination [35]. Such changes in rates with temperature are equivalent to an activation energy for the pre-exponential factor of ∼110 kJ/mol, which is 3-fold larger than expected for a 40-residue fast-folding protein [17]. The thermodynamic analysis described in the previous section points to coupling between BBL unfolding and proton transfer as a putative source for this unusual behavior. Proton transfer processes between water and amino-groups are controlled by the bi-molecular collision rate between the proton donor and acceptor [48], and thus become relatively slow around neutral pH where the total concentration of H_3_O^+^ plus OH^−^ is lowest. For instance, at neutral pH and room temperature, the proton transfer rate for the arginine side-chain measured by magnetization-transfer NMR experiments is ∼ 1200 s^−1^ [49]. Moreover, proton transfer rates display significant temperature dependence [48] [49]. Therefore, at neutral pH the overall folding-unfolding kinetics of BBL in the presence of chemical denaturants could be controlled by protonation-deprotonation of a single basic residue, especially at the low temperatures employed in the SM-FRET experiments.

To investigate this hypothesis, we looked into the BBL folding-unfolding relaxation kinetics near the denaturation midpoint over the pH range in question. To achieve the appropriate time-resolution and dynamic range we used the laser-induced temperature jump technique implemented on a pump-probe configuration. This configuration allows easy acquisition of kinetic data in logarithmic time from nanoseconds to milliseconds [11]. As probe of native structure in BBL we used fluorescence Förster energy transfer (FRET) between the pair of dyes Alexa 564 (A564) and Alexa 647 (A647) as donor and acceptor, respectively. These dyes were incorporated onto cysteines added to the termini of a BBL sequence that also includes 4-residue flexible tails [32]. The advantage of this approach is that it replicates the previous SM-FRET measurements [32]. We performed laser T-jump experiments in which we induced jumps of ∼5 K to a final temperature of ∼280 K (i.e. similar to that of the SM-FRET experiments) at concentrations of GdmCl near the BBL denaturation midpoint for each pH value within the 6–11 range. The experiments were done at the denaturation midpoint because under these conditions BBL has the same stability across the pH range and thus the folding kinetics should be equivalent. Moreover, it is also under these conditions that the changes in protonation are expected to be largest in magnitude if there is indeed strong coupling between folding and proton transfer.

**Figure 3:**
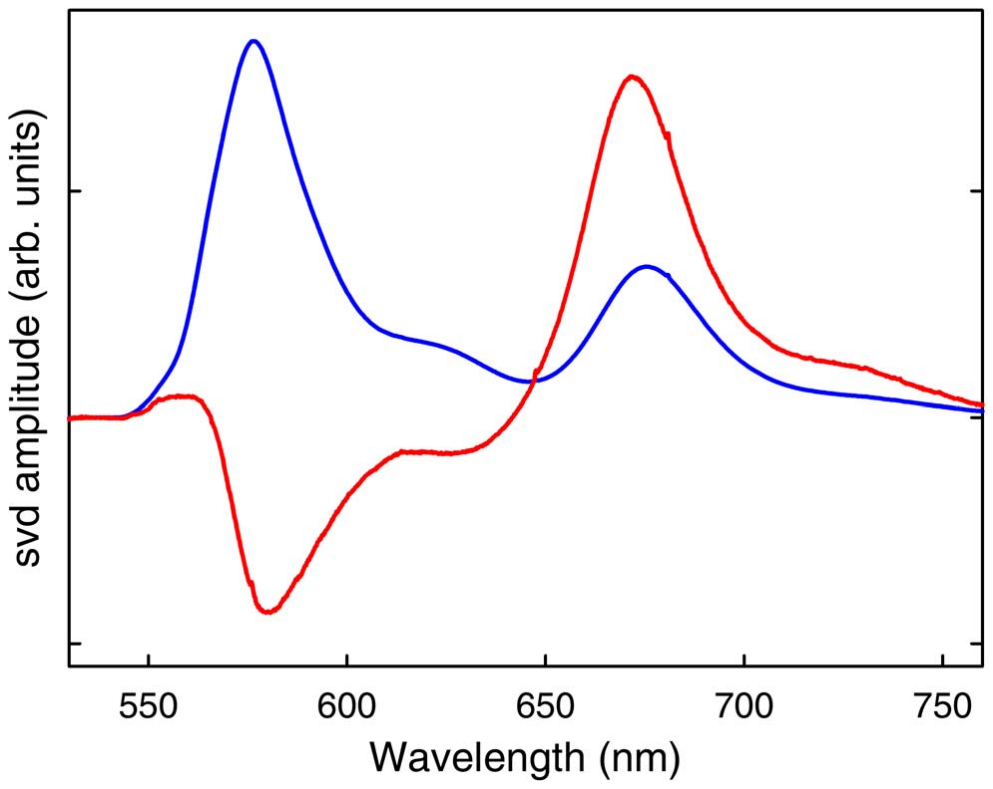
Main components from the singular value decomposition (svd) of the laser-induced T-jump experiments of BBL labeled with A564 and A647. The first component (blue) shows the average fluorescence spectrum for all times at all pH values. The second component (red) shows the time-dependent changes in fluorescence.

**Figure 4:**
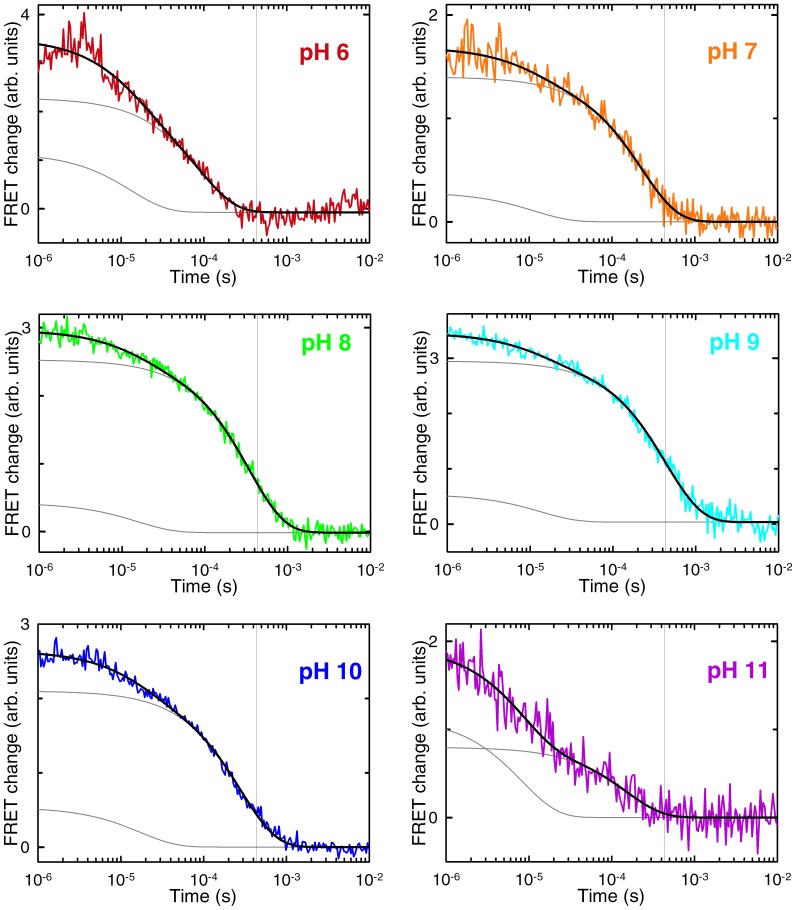
Folding-unfolding relaxation kinetics of BBL at the chemical denaturation midpoint after T-jumps of ∼ 4 K to a final temperature of 279 K. The panels show the experimental decays at the various pH values (colored according to the code of previous figures), as obtained from the amplitude of the second svd component (red in Fig. 3). The black lines are fits to a double exponential function. The individual exponential decays obtained from the fits are shown in grey. The vertical grey line signals the relaxation time for the slow phase at pH 9.

**Figure 5:**
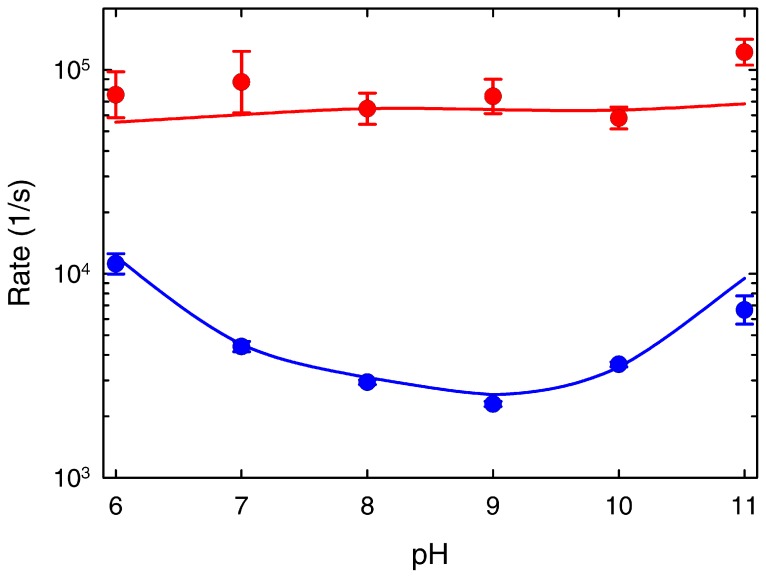
pH dependence of the two phases observed in the T-jump experiments of BBL at 279 k near the chemical denaturation midpoint. The fast phase is shown in red and the slow phase in blue. The error bars signify the fitting errors to the double exponential fits of Fig. 4. The blue and red curves are the predictions by the 1D free energy surface model (see Fig. 6).

The results from such experiments are summarized in Figs. 3–5. We performed a global singular value decomposition (SVD) analysis of the matrix containing the whole experimental dataset of time-dependent fluorescence spectra of BBL at the various pH values. This procedure rendered two main components (Fig. 3). The first component is the average spectrum of the BBL samples at all pH values, showing the fluorescence emission of the donor (A564) and acceptor (A647). The second component shows an anti-correlation between fluorescence emissions of the two dyes, indicating a change in FRET signal during the kinetic experiments (red in Fig. 3). The amplitude of the second SVD component renders the kinetic decays at the different experimental pH values (Fig. 4). All the kinetic decays show a decrease in FRET as BBL is heated from 275 to 280 K near the chemical denaturation midpoint; i.e. the overall change in amplitude goes from positive values to zero, which (when multiplied by the spectral signature of the second svd component shown in red in Fig. 3) represent reduction in acceptor emission coupled to increase in donor emission. The kinetic decays exhibit clear pH dependence even though they have been measured in conditions of iso-stability for BBL. Another interesting observation is that all the decays, which are measured in logarithmic time from 1 µs to 10 ms, are markedly non-exponential. In fact they can be best fit to a double exponential function (black curves in Fig. 4). This is an important result because this time window does not include the nanosecond timescales characteristic of the conformational motions of unfolded polypeptides [16], which are also observed in FRET T-jump experiments of proteins with flexible tails [28].

The two observed kinetic phases are thus directly connected to the folding-unfolding relaxation of BBL. According to the bi-exponential fits, the fast phase is essentially pH independent with a rate of ∼1/(15 µs) for all conditions. In contrast, the slow phase goes from a maximal rate of ∼1/(90 µs) at pH 6 down to a minimum of ∼1/(435 µs) at pH 9, speeding up again to ∼1/(150 µs) at pH 11. The overall pH dependence of the BBL folding relaxation kinetics can be observed by comparison to the vertical lines in the figure panels (which signal the slowest relaxation time). It is also noteworthy that the slow rate that we measure at pH 8 (i.e. 1/(340 µs)) is identical to that previously reported by Fersht and coworkers [33], further confirming our previous assertion that their experimental conditions correspond to pH 8 [35]. The specific pH-induced changes in the rates for the fast and slow phases determined from the bi-exponential fits are shown in Fig. 5. The relative amplitudes also exhibit pH dependence (see the fitted fast and slow decays for each pH value as grey thin curves in the Fig. 4 panels). Particularly, the slow phase has maximal amplitude in the pH 7–9 region, where it amounts to ∼85% of the total amplitude, and decreases at both pH extremes.

**Figure 6:**
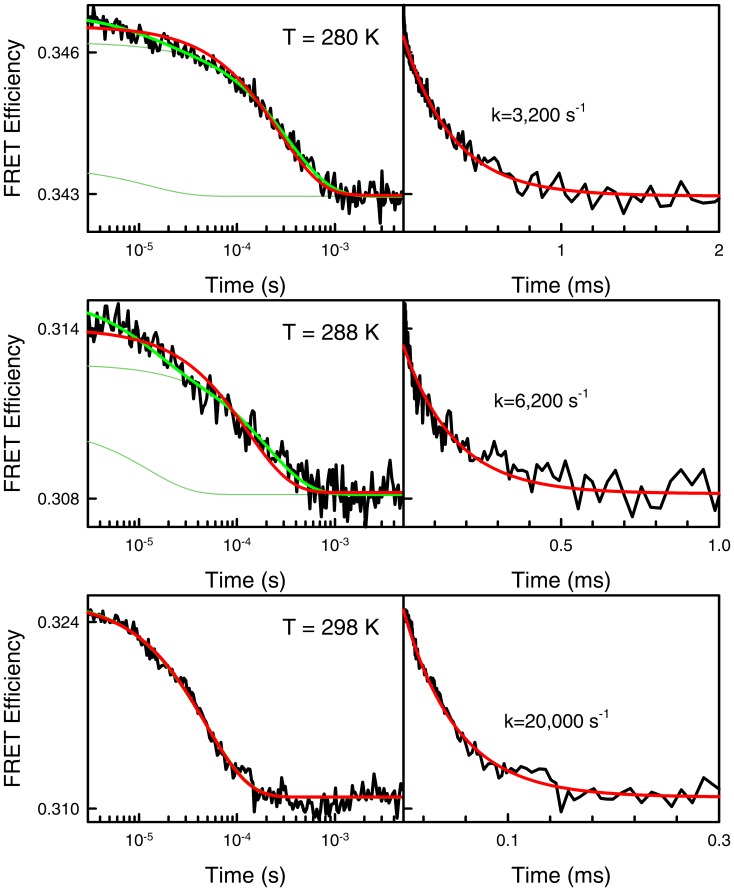
T-jump folding-unfolding relaxation kinetics of BBL at various final temperatures measured at pH 8 and near the chemical denaturation midpoint. The left panels show the experimental decays measured in logarithmic time and fitted to double (green) and single (red) exponential functions. The right panels show the same experimental data plotted in linear time and fitted to a single-exponential function.

All these observations point to kinetics controlled by the interplay between folding-unfolding and protonation-deprotonation of a residue titrating around pH 7. The observation of biphasic kinetics for BBL under the conditions used for the SM-FRET experiments reveals an underlying complexity that contrasts with the single-exponential microsecond kinetics previously observed in laser T-jump experiments performed by several authors at high temperature [28] [50]. Slower, but still apparently single-exponential kinetics have been also reported by the same groups at room temperature near the chemical denaturation midpoint [28], [50]. Moreover, previous laser T-jump experiments performed at the same conditions used here and pH 6 [32] or pH ∼8 [33] also produced apparently single exponential decays. This discrepancy is puzzling. The main difference is that previous kinetic decays were acquired in linear time rather than logarithmic. It is thus possible that the linear-time kinetic experiments could not resolve the ∼1/(15 µs) fast phase with the limited signal to noise ratio of T-jump experiments. The fast phase could be particularly hard to resolve at low temperature where the slow phase is up to 30-fold slower. To investigate this possibility we measured T-jump kinetics for the pH 8 sample around the C_m_ and between 280 K and room temperature (Fig. 6). As expected, at 298 K the overall relaxation becomes much faster (i.e. 1/(50 µs), in excellent agreement with the rates determined before at equivalent conditions [28], [50]). The room temperature kinetic decay is also essentially single exponential, even when measured in logarithmic time (bottom left panel in Fig. 6). On the other hand, the relaxation decay at lower temperature is distinctly double exponential in logarithmic time, but it is well fit to a single exponential function with the same relaxation time of the slow phase when measured in linear time (top and middle panels in Fig. 6).

The experiments shown in Fig. 6 confirm that our current data is compatible with all previous T-jump data and buttress the existence of two phases in the folding-unfolding kinetics of BBL. The fast phase exhibits a pH independent rate that coincides with the folding timescales expected for downhill folding proteins [17]. The parabolic pH dependence of the slow phase points to a process limited by a slow proton transfer step that exhibits acid- and base-catalysis [48]. Such pH dependence is much weaker than that measured for proton transfer rates in free amino acids [49], presumably because the multimolar GdmCl solutions act as efficient proton exchange catalyst [48]. The sub-millisecond rates of the slow phase are also comparable to the proton transfer rates expected for amino groups around neutral pH [48], [49]. Likewise, the surprisingly strong temperature dependence observed for the BBL folding relaxation kinetics in this pH range could reflect a switch between a high-temperature regime in which proton transfer is faster or comparable to the folding-unfolding rate, and a regime occurring at low temperatures in which the proton exchange step becomes rate limiting.

### A Simple Theoretical Model for Downhill Folding Coupled to Proton Transfer

The equilibrium and kinetic results discussed in the previous two sections demonstrate empirically that the unfolding of BBL within the pH 6–11 range is tightly coupled to a specific proton exchange process. However, at this point it is very important to explain these results at a quantitative level and ascertain whether they are compatible with the prior battery of data and analyses that have identified BBL as a one-state downhill folder. Ultimately, we would like to rationalize the stark differences between the SM-FRET results produced on this protein by us (a unimodal distribution of partly unfolded molecules) [32], and the Fersht group (a bimodal distribution at the denaturation midpoint) [33].

Those goals are best approached analyzing the experimental data with simple, non-committal theoretical models. In this case we have modified our previously developed one-dimensional free energy surface model of protein folding to incorporate the equilibrium and kinetic effects of protonation-deprotonation. This model is based on the energy landscape approach [51] and directly accounts for size-scaling effects in protein folding [14]. In spite of its analytical simplicity, the model has proven to be extremely effective for interpreting a vast array of protein folding experiments quantitatively. For instance, it was instrumental for the interpretation of deviations from two-state behavior in the folding relaxation kinetics of fast-folding proteins [17], for extracting thermodynamic folding barriers from differential scanning calorimetry data [39], and for the global quantitative analysis of equilibrium and kinetic folding data in general [37], [38], as well as the characterization of the one-state downhill folding regime in the protein BBL based on multiprobe T-jump kinetic data [28] and, ultimately, on single-molecule FRET experiments [32]. Moreover, the model has shown considerable predictive power, which has been demonstrated by predicting protein stability and downhill folding behavior from protein size [40], and by predicting absolute protein folding and unfolding rates using size and structural class (total of 10-bits) as only protein-specific input [41].

We have thus taken this model and modified it to incorporate the coupling between folding and protonation-deprotonation of a single titrating group. The detailed description of these model modifications is provided in the Experimental Methods and Theoretical Model section. Briefly, the model splits the discretized one-dimensional free energy surface as a function of the characteristic order parameter (i.e. nativeness) onto two different surfaces, corresponding to the protonated and unprotonated forms of the protein. The free energy surfaces for the two forms are determined by the overall BBL energetic parameters and simple ionization equilibria relationships between the equivalent microstates (equal nativeness) as defined by specific *pK_a_* values. Coupling between overall folding and ionization results from the difference between the weighted average *pK_a_* for the microstates defining the native and the unfolded ensembles.

For simplicity, we defined a linearly decreasing dependence of the *pK_a_* with the order parameter. Particularly, we chose a scale that goes from effective *pK_a_* values of ∼11 for the fully unfolded ensemble (i.e. <nativeness> = 0.3) to ∼6 for the native ensemble (<nativeness> = 0.9). This range in *pK_a_* is consistent with an arginine or lysine residue that experiences a large shift of its ionization equilibrium towards the unprotonated state in the native 3D structure. Once implemented with such straightforward description of folding coupled to binding the model generates a pH titration curve in the absence of chemical denaturants that nicely follows the trend of the experimental data (gray line and left scale in Fig. 2). Here it is important to mention that the changes in mean nativeness as function of pH produced by the model correspond to a one-state shift of the single BBL “native-like” ensemble to less degree of structure in response to the lower pH, rather than a conversion between folded and unfolded states. The calculated native signal shows a plateau at pH ∼5 because the model does not include the ionization of additional BBL residues. We omitted additional ionization equilibria for the sake of simplicity since they are not central to the behavior across the pH 6–11 range that we seek to understand.

The kinetic implementation of the model only requires defining a set of kinetic transitions connecting the protonated and unprotonated forms of the protein. Here we followed the most conservative definition in which proton transfer occurs only between structurally identical microstates (i.e. species of same nativeness). We thus defined a rate matrix that treats the conformational dynamics on the protonated and unprotonated surfaces as diffusive, and connects the two surfaces by proton exchange on- and off-rates between equivalent microstates (i.e. equal nativeness). The only new parameters are the on- and off-rates for the proton exchange reactions. We defined the microscopic proton exchange on-rates as the sum of three second-order microscopic rate constants to account for: 1) acid catalysis (proportional to [H^+^]), 2) base catalysis (proportional to [OH^−^]), and 3) amino-transfer processes (here proportional to [guanidinium]) [48]. Proton release (off) rates are then directly determined by detailed balance according to the *pK_a_* for each nativeness microstate. To simulate the kinetic experiments we just needed to find values for the three microscopic proton exchange on-rates (the acid-, base- and amino-transfer rates) that agree with the experimental observations (see the Experimental Methods and Theoretical Model section).

**Figure 7:**
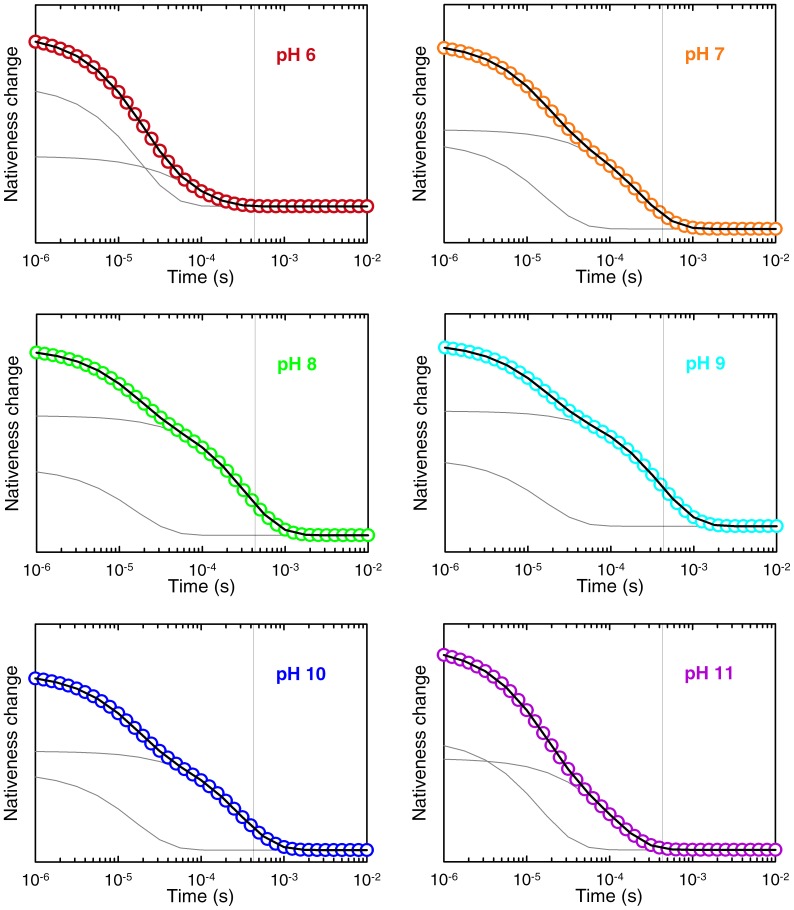
Folding-unfolding relaxation kinetics of BBL as predicted by the 1D free energy surface model implemented with the unfolding coupled to proton transfer mechanism. The panels show the time-dependent changes in mean nativeness as a function of pH. Color code and curves as in Fig. 4.

Using the parameterized rate matrix we simulated the T-jumps experiments near the denaturation midpoint of Fig. 4. In these simulations we integrated the time-dependent probabilities for all states in the model to compute the average nativeness as a function of time (note that the probability for each value of nativeness is the sum of the protonated and unprotonated forms). This calculation can be directly compared to experiments that use a structural probe that changes linearly with the degree of folding. That is in fact not an unreasonable assumption for describing the FRET signal between donor and acceptor placed at the protein ends used in our experiments of Fig. 4. However, we should also emphasize that we are not interested in fitting the experimental signals exactly, since this effort would involve using additional (not well defined) parameters to describe the end-to-end distance for each microstate. Our goal is simply to compare experiment and theory at a quantitative level, but in a most general way. Nevertheless, the results of the T-jump simulations reproduce the experimental data very closely. The simulated decays are biexponential at all conditions (Fig. 7), with a fast phase that is pH independent and a slow phase with the characteristic experimental pH dependence (lines in Fig. 5). As seen before experimentally, the amplitude of the slow phase is maximal between pH 7 and 9, whereas the fast phase becomes more prevalent at the extreme pH values. There is no exact agreement between simulation and experiment in terms of the relative amplitudes for the two phases. Particularly, the simulation tends to produce slightly larger amplitude for the fast phase (compare grey lines in Figs. 4 and 7). However, we could not realistically expect better agreement given that we are directly comparing the decay of the model order parameter with an experimental FRET signal.

**Figure 8:**
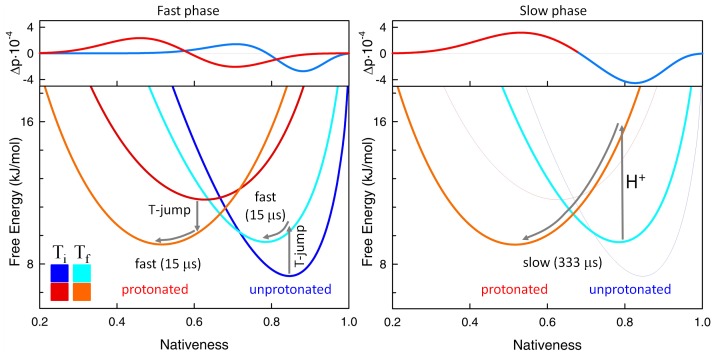
Interpretation of the biphasic folding-unfolding kinetics of BBL. The figure shows the calculations with the FES model of a T-jump relaxation to near midpoint denaturation conditions at pH 8. The bottom panels show the free energy surfaces for the protonated and unprotonated forms before (darker colors) and after (lighter colors) the T-jump. The top panels show the changes in probability associated with each phase, as obtained from the eigenvectors of the rate matrix. Red shades signal the protonated species and blue shades signal the unprotonated species. The proton transfer step is shown with an arrow plus the symbol H^+^.

The general consistency between simulations and experiment provides a straightforward interpretation of the biphasic pH-dependent exponential kinetics of BBL (Fig. 8). According to the model, the 1/(15 µs) phase corresponds to the downhill unfolding relaxation of the unprotonated and protonated populations in response to the nanosecond T-jump (left panel in Fig. 8). The increase in temperature tilts the folding free energy surface of both protonation states towards more unfolding so that each protonation species relaxes diffusively towards its new free energy minimum. The slow pH-dependent phase involves the reequilibration between the unprotonated and protonated forms induced by the increasing temperature. It is triggered by a sub-ms proton transfer reaction, followed immediately by the diffusive relaxation of the newly protonated molecules to their (more disordered) free energy minimum (right panel in Fig. 8). Therefore, the biphasic kinetics we observe for BBL seem to be a consequence of strong coupling between proton transfer and the gradual disordering of downhill folding proteins.

### Explaining Unfolding Heterogeneity in Single-Molecule Fluorescence Experiments of BBL

In the previous sections we demonstrated that BBL folding-unfolding is thermodynamically and kinetically coupled to the specific protonation of a yet unidentified basic residue that takes place in the pH 6–11 range. Thus denaturing agents (e.g. GdmCl) induce protonation, and lower pH favors BBL unfolding. Moreover, in that pH range and at ∼280 K, proton transfer happens to be slow (Figs. 4–5). An important remaining issue is whether the coupling between one-state downhill folding and the binary (and rate limiting) proton exchange process could explain the seemingly confronting observations on the chemical unfolding of BBL made by different groups using equivalent single-molecule fluorescence methods.

**Figure 9:**
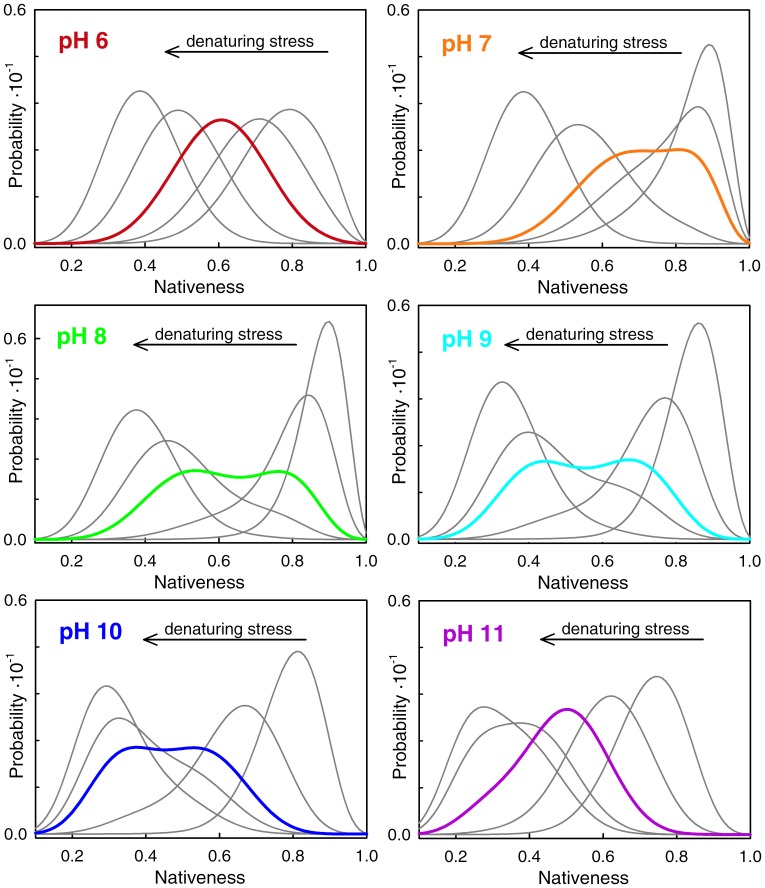
Conformational ensemble of BBL as a function of pH and denaturing stress as predicted by the 1D free energy surface model. Chemical denaturation was simulated as explained in the Experimental Methods and Theoretical Model section. The panels show the conformational ensembles for five conditions from highly native to highly denaturing at each pH value (color code as before).

To address this question we looked into the unfolding behavior at the single-molecule level predicted by the one-dimensional free energy surface model. Simulations of BBL unfolding at different pH values show two very distinct regimes (Fig. 9). Interestingly, the two regimes are best represented by the two pH conditions tested in SM-FRET experiments. At pH 6 (conditions for the experiments of Liu et al [32]) BBL exhibits the characteristic unimodal one-state downhill behavior; namely BBL populates a single ensemble at all conditions including the midpoint, but the ensemble shifts gradually from native to unfolded values of the order parameter as denaturing stress increases (top left panel in Fig. 9). However, around pH 8 (the true experimental conditions of Huang et al [33]) the unfolding behavior of BBL is distinctly bimodal, being most apparent at the denaturation midpoint, where the model produces two partially overlapping conformational ensembles with maxima at 0.75 and 0.5 nativeness (middle left panel in Fig. 9). The “native” and “unfolded” ensembles inter-convert in response to denaturing stress. But, in addition, the two ensembles display gradual unfolding (shifts to lower values of the order parameter) as stress increases. At pH 7, the model produces somewhat intermediate behavior in which the two peaks overlap more although are still distinguishable (top right panel in Fig. 9). At the highest pH values BBL turns back to a single gradually shifting ensemble with a unimodal distribution at the midpoint (bottom right panel in Fig. 9). Therefore, the model reproduces the two behaviors observed experimentally, and postulates a pH-dependent shift between the two regimes, which is incidentally supported by SM-FRET data obtained by Huang et al. close to pH 7 (see the marginally bimodal distribution obtained at the midpoint using urea as denaturant at pH 7, sup. mat. in [33]).

**Figure 10:**
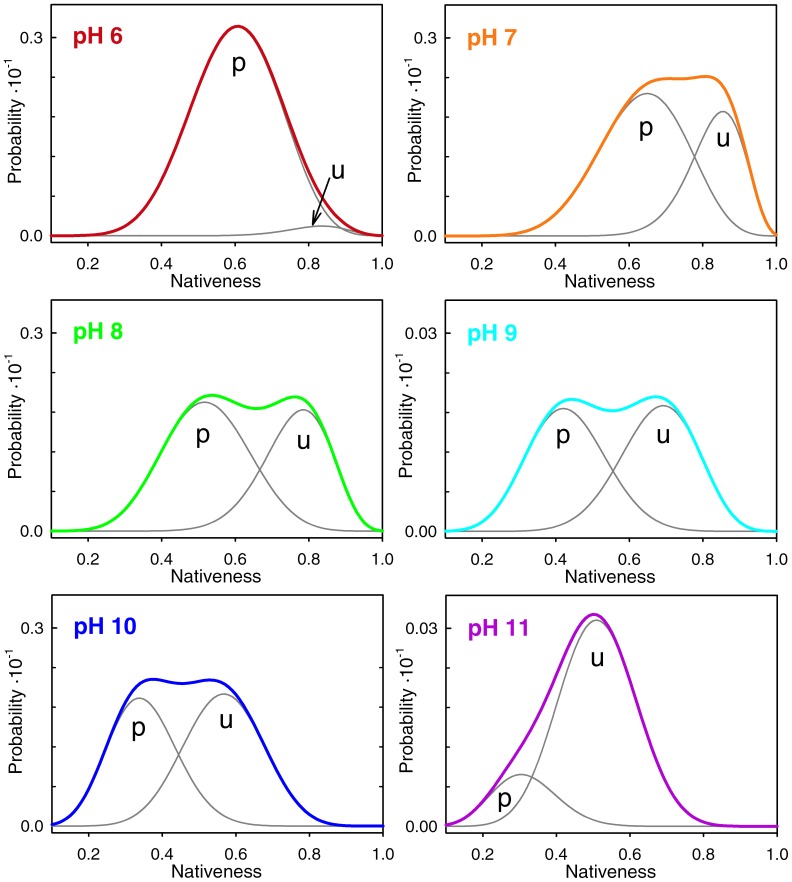
Conformational ensemble of BBL at the denaturation midpoint as a function of pH predicted by the 1D free energy surface model. The grey curves indicate the contribution of the protonated (p) and unprotonated (u) forms of the protein to the overall conformational ensemble.

Beyond the ability to reconcile two apparently conflicting experimental observations, these theoretical calculations shed light onto the physical mechanism underlying the switch between the unimodal and bimodal regimes in BBL. This is an important issue because the most direct interpretation of the pH-dependent switch would be that protonation induces a transition from two-state to one-state downhill folding. This explanation would go along the lines of what has been proposed before based on coarse-grained simulations of protein folding [24]. However, what our analysis shows is that BBL switches from a unimodal distribution to a bimodal one without really changing its underlying folding mechanism. The bimodal regime arises from the splitting of its single conformational ensemble onto two one-state sub-ensembles that differ in their protonation status and thus on their intrinsic stability. As illustration of this point, Fig. 10 shows the BBL conformational ensemble near the denaturation midpoint differentiating between the protonated and unprotonated forms of the protein. This figure shows that the two peaks that are apparent near the denaturation midpoint within the pH 7–10 range correspond to the protonated and unprotonated species of the protein, and not to conventional native and unfolded states. The two species are resolved in the single-molecule fluorescence experiments because they are structurally distinct (e.g. different degree of nativeness) and their inter-conversion by proton exchange is rate limiting. However, and most critically, the two BBL species unfold gradually in parallel to their inter-conversion by proton exchange (see the shifting peaks in the equilibrium distributions of Fig. 9). Thus both species adhere to the one-state downhill scenario, as it can be clearly appreciated in the free energy surfaces at pH 8 near the denaturation midpoint obtained by the parameterized model (Fig. 8). In fact, the special properties of the one-state downhill scenario are what make the two protonation species structurally different: the protonated form is intrinsically less stable and, as such, it is less structured at any given experimental condition. Summarizing, slow proton exchange coupled to folding splits the one-state downhill folding ensemble of BBL onto two species that differ chemically (with and without one proton) and structurally. Both species unfold gradually in one-state downhill fashion due to the action of denaturing agents. Moreover, because the denaturing agents favor the more unstructured protonated form of BBL, the denaturation experiment also induces the binary conversion between the two species.

**Figure 11:**
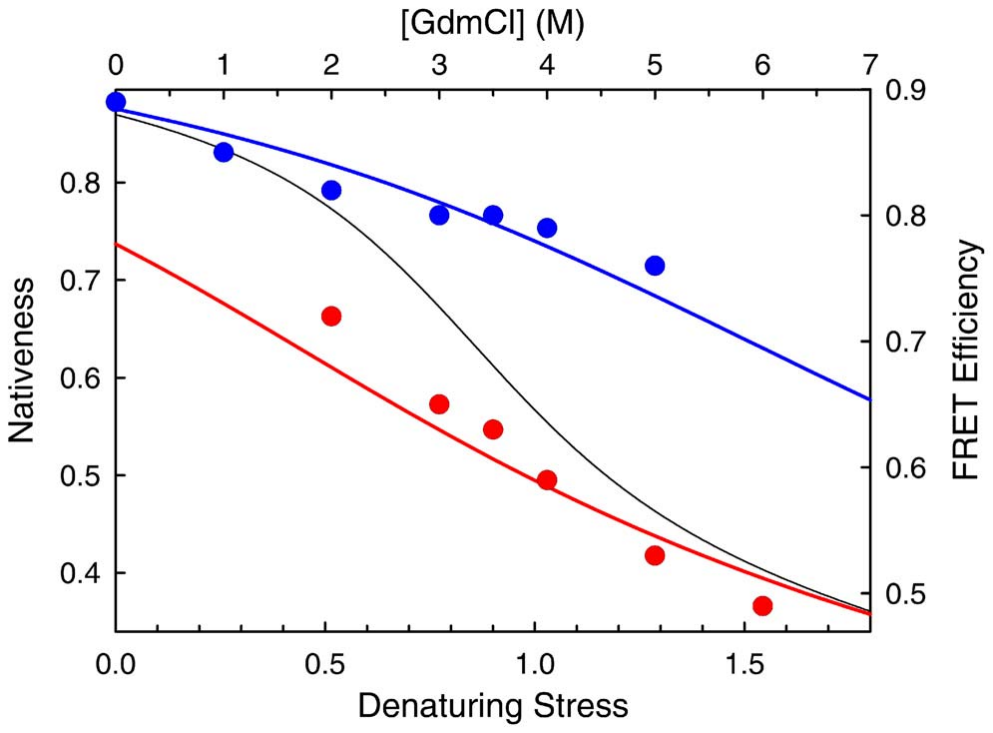
Gradual unfolding of the two peaks observed in the chemical denaturation of BBL at pH 8. The circles, right and top axes show the experimental FRET maxima obtained by Huang et“native” (blue) and “unfolded” (red) peaks. The colored curves, left and bottom axes show the changes in nativeness as a function of denaturing stress (the value at the maximum) predicted by the 1D free energy surface model for the unprotonated (blue) and unprotonated (red) forms of BBL. The black curve is the predicted mean changes in nativeness.

Interestingly, the coupling between a rate-limiting proton transfer event and the one-state downhill folding scenario nicely explains several intriguing features of the Huang et al. SM-FRET experiments. In those experiments, the two detected peaks (i.e. the “native” and “unfolded” states) exhibited marked, denaturant-induced decreases in *E* suggestive of expansion and/or disordering. This observation is now common place for the unfolded ensemble of slow two-state folding proteins [30], and signals its swelling by the action of chemical denaturants [52]. However, a similar expansion is not observed for the peak corresponding to the native state in these proteins, in line with the expectation for a true two-state model [53]. On the other hand, the one-state scenario coupled to proton transfer does naturally produce two peaks that gradually unfold in parallel to their inter-conversion induced by denaturants. In fact, the shifts in *E* observed experimentally by Huang et al. are very similar to the theoretical predictions of the one-state downhill scenario coupled to proton transfer for BBL. Fig. 11 compares the experimental shifts with the gradual unfolding of the non-protonated and protonated species predicted by the model. The coupling between one-state downhill chemical unfolding and protonation also explains the large discrepancy in *C_m_* between the SM-FRET experiments (<3.5 M) and the bulk CD unfolding curve (∼4.2 M) in the Huang et al. experiments [33]. The explanation is straightforward since the theoretical model suggests that the two experiments measure different things. The SM-FRET *C_m_* is obtained by integrating the areas of the two peaks, which according to the model report on the populations of the protonated and non-protonated species, rather than of “native” and “unfolded” states. In contrast, the *C_m_* from the bulk CD curve signals the denaturant concentration at which the overall change in secondary structure is halfway. For BBL this property would include the compounded effects from the gradual unfolding of each species plus their inter-conversion.

**Figure 12:**
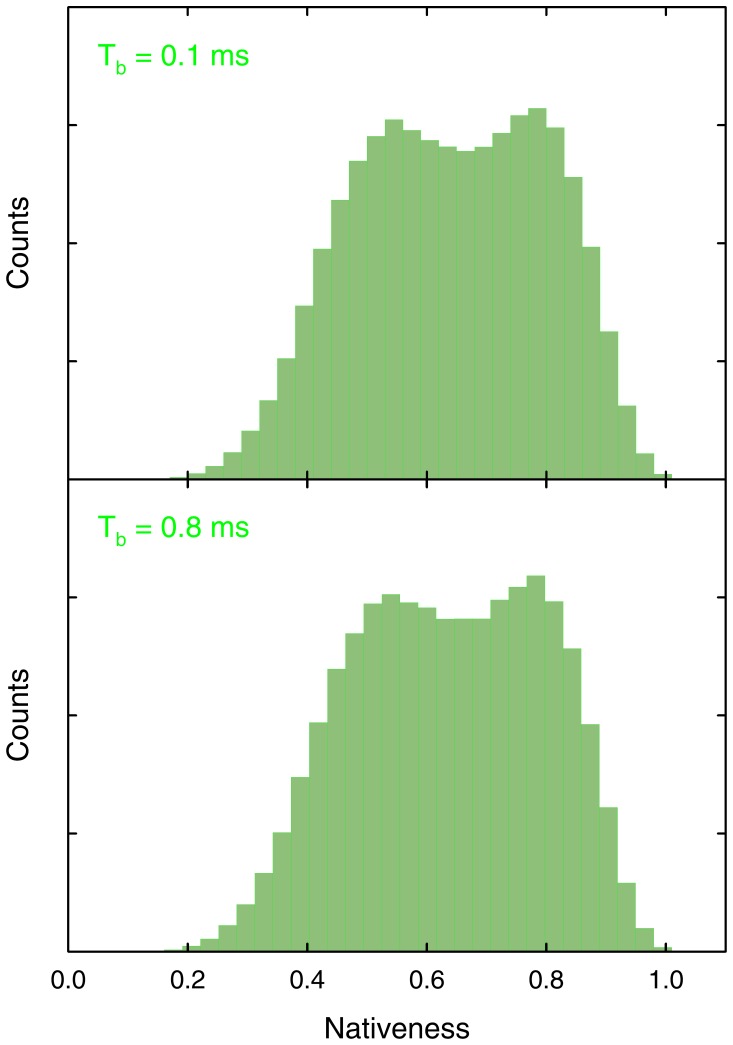
Single molecule histograms at the pH 8 denaturation midpoint for BBL calculated for two different binning times using stochastic kinetic simulations with the 1D free energy surface model.

Finally, the clear pH dependence of the slow conformational rate in BBL (blue in fig. 5) explains why Huang et al. still observed a bimodal distribution in the SM-FRET histograms obtained with comparatively very long binning times (i.e. 0.8 ms) [33]. At lower pH the slow rate is sufficiently fast to produce dynamic conformational averaging over the 0.8 ms bins. Accordingly, we argued that a midpoint histogram obtained with 0.8 ms time bins should show a broader single peak rather than the two peaks obtained with shorter binning times [36]. However, at the actual pH ∼8 of the Huang et al. experiments [35] the proton transfer rate is in fact significantly slower (Fig. 5). Such slow down implies that 0.8 ms is still sufficiently short so that dynamic averaging is not dominant and the two protonation species of BBL can be resolved. This point is easily demonstrated by simulating the single-molecule histogram expected at pH 8 and 0.8 ms binning times using the stochastic kinetic simulations with our theoretical model (Fig. 12).

## Conclusions

The observation of both unimodal and bimodal distributions during the chemical denaturation of the protein BBL investigated with SM-FRET poses an intriguing conundrum. Unimodal distributions are expected for a protein that has been categorized as a one-state downhill folder according to an extensive battery of quantitative tests. On the other hand, the observation of two-peaks demonstrates that there are two inter-converting states in the chemical unfolding of BBL. The questions that emerge are: what are those two states? And why are these two states not seen in SM-FRET experiments with higher resolution under slightly different experimental conditions? Our premise was that these apparently incompatible observations could originate in the coupling between BBL unfolding and a specific proton transfer process. The experiments and analysis reported here demonstrate the presence of such coupling and provide an integral explanation of all the available experimental data on BBL.

For instance, our thermodynamic chemical denaturation experiments at different pH values indicate that BBL unfolding is tightly coupled to the protonation of one of its residues with an apparent *pK_a_* of ∼7. Consequently, the destabilization of the BBL native state (e.g. by adding chemical denaturants) within the pH 6–11 range results in both unfolding and protonation. In other words, the overall BBL unfolding process is accompanied by the conversion from the unprotonated to the protonated species. Laser-induced T-jump experiments at the denaturation midpoint show that the relaxation kinetics of BBL is complex (bi-exponential) and pH dependent. Within the pH 6–11 range and at the low temperature of previous SM-FRET experiments (∼280 K), we find that proton exchange is much slower than the folding-unfolding kinetics of BBL. The proton transfer step thus determines the timescale of the overall relaxation process, which becomes rather slow (down to ∼ 2000 s^−1^ at pH ∼9).

These observations bear deep implications for the interpretation of single-molecule experiments of BBL chemical unfolding. The coupling between unfolding and proton transfer splits the conformational ensemble of BBL onto two chemically heterogeneous species that inter-convert slowly. The strong coupling also makes their inter-conversion dependent on both pH and chemical denaturant, thus being an obvious source for the bimodal distributions observed at pH ∼8. There are, however, two alternative interpretations in terms of the underlying folding mechanism. One possibility is that above neutral pH BBL unfolds in a conventional two-state fashion so that both chemical denaturants and protonation stabilize the fully unfolded state. In this case, the slow proton transfer step at low temperature would only act as a kinetic retardant thus making it feasible to resolve the “native” and ”unfolded” states with the limited time resolution of SM-FRET experiments. The problem is that such two-state interpretation is inconsistent with the observation of bi-exponential kinetics (Fig. 6), and the fact that the pH-independent fast phase coincides with the relaxation time measured before for the overall folding-unfolding process of BBL at room temperature in the absence of chemical denaturants [28]. This interpretation would also require a switch from the two-state scenario at pH 8 to a one-state scenario at pH 6 given that the distinctly unimodal SM-FRET histograms observed at the latter pH cannot be explained as two unresolved peaks [32]. Furthermore, the two-state interpretation is inconsistent with a battery of thermodynamic and kinetic experiments that have been performed at neutral pH and support the one-state downhill scenario for BBL [18], [19], [22], [28].

An alternative interpretation is that BBL folds-unfolds in a one-state downhill manner at all conditions. Under this scenario, both species (unprotonated and protonated) unfold gradually. Their inter-conversion is induced by chemical denaturants and results in bimodal distributions because at each experimental condition the protonated species is relatively more disordered. Using statistical mechanical modeling we show here that this scenario reproduces quantitatively all the thermodynamic and kinetic data on BBL obtained over the pH 6–11 range. The coupling between proton transfer and one-state downhill folding also reproduces the unimodal distributions observed by SM-FRET at pH 6 [32] together with the emergence of bimodal SM-FRET histograms between pH 7–8, as Huang et al. observe [33]. Even more strikingly, this simple mechanism can also explain the unconventional features of the Huang et al experimental results: 1) the fact that the peak labeled as native state exhibits large decreases in *E* as a function of denaturant; 2) the discrepancy between the *C_m_* measured by bulk CD and SM-FRET; and 3) the lack of dynamic averaging in the histogram obtained using 0.8 ms time bins.

Summarizing, our experiments and analysis show that a conventional two-state folding mechanism cannot explain the thermodynamic, kinetic and single-molecule behavior of BBL in the pH 6–11 range. Of course, it is always difficult to rule out more complex interpretations that resource to multiple intermediate states in BBL. However, what we can unambiguously conclude is that the coupling between a slow proton transfer step and one-state downhill unfolding is the simplest mechanism that explains all the available experimental data on BBL folding at the quantitative level, and thus solves the puzzle of the apparent inconsistency in single-molecule experiments.

## Experimental Procedures and Theoretical Calculations

### Preparation of BBL Samples in Multimolar GdmCl Solutions

For all the experiments performed under this study we used the same long-variant of BBL including short unstructured tails that we have used before for SM-FRET experiments [32]. To measure the chemical denaturation of BBL at fixed pH values we took into consideration the effects of the ionic chaotrope GdmCl on the pH reading of glass electrodes, as well as the *pK_a_* shifts of the biochemical buffers induced by the highly increasing ionic strength associated to multimolar concentrations of GdmCl [35], [44]. We guarantee that all samples of a given denaturation curve were set to the same pH value regardless of GdmCl concentration by adjusting the pH of each sample individually. Particularly, we adjusted the pH of each sample by mixing GdmCl solutions at the target final concentration prepared on the acid- and the base- components of the buffer. The mix was varied until obtaining the target pH reading from the glass electrode of a standard pH-meter. We calculated the target pH reading as a function of GdmCl using the empirical correction formula obtained by Garcia-Mira and coworkers [44]. We then prepared the final solution mixing the pH-adjusted buffer (to a final concentration of 50 mM) with a highly concentrated stock solution of BBL (to a final concentration of 50 µM), and NaCl to reach an ionic strength of 250 mM (without considering the contribution from GdmCl). The pH and the concentration of GdmCl of each sample were rechecked after the experiments with a glass-electrode and by refractometry, respectively. For the experiments at pH 6 and 7 we used phosphate buffer, for pH 8 MOPS buffer, for pH 9 we used borate buffer and for pH 10 and 11 carbonate buffer. The BBL samples for CD experiments were prepared at a protein concentration of 50 µM. The samples for the fluorescence T-jump experiments were prepared at a protein concentration of 10 µM using samples of BBL labeled with Alexa546 and Alexa647 as donor and acceptor probes for FRET measurements. The samples for T-jump experiments were prepared at concentrations of GdmCl roughly coinciding with the chemical denaturation midpoint at each pH and adding 5 mM ascorbic acid to minimize photodamage of the fluorescent probes.

### Circular Dichroism Denaturation Curves

CD spectra were acquired on a Jasco J-815 spectropolarimeter equipped with a Peltier temperature controller to keep the temperature of the sample fixed at 279 K.

### Fluorescence T-Jump Experiments

FRET laser-induced temperature jump measurements were performed using a custom built apparatus based on a previous setup [11]. Briefly, the fundamental wavelength of a Nd:YAG laser (Litron Nano-LG-320) operating at a repetition rate of 1 Hz is shifted to 1907 nm to match the absorption of the vibrational modes of water using a 1 m path Raman cell (Lightage inc.) filled with a mixture of H_2_ and Ar at 1500 psi. The first Stokes converted beam is selected using a Pellin-Broca prism and an iris, and then focused onto the sample. In this way heating pulses up to 20 mJ were obtained, generating local temperature jumps of about 8–10 K. The response of the sample to this perturbation was observed using as a probe the fluorescence emitted by the attached dyes. Donor (Alexa 546) excitation was achieved via the second harmonic (532 nm) of a Nd:YAG laser (Ekspla NL202/SH). The fluorescence emitted by the sample was collected perpendicularly with respect to the excitation beam path using a plano-convex lens, dispersed via a spectrograph (Princeton Instruments Acton SpectraPro 2150i) and spectra were recorded using a back-illuminated CCD camera (Princeton Instruments Pixis 100). The delay between the pump and probe lasers were set using a digital delay generator (Stanford Research Systems DG535) and measured by a digital counter (Agilent technologies 53132A), with a jitter between the two pulses of about 1 ns. Spectra at different delay times were accumulated and analyzed using a Matlab custom script to obtain the kinetics. The sample solution was held in a quartz cuvette with detachable windows having an optical path of 0.5 mm and thermostated at the proper base temperature using two Peltier thermoelectric coolers (TE Technology inc) in a custom-built sample holder. The amplitude of the temperature jump was calibrated using Rhodamine B as a standard. After calibration, we performed the set of measurements setting the protein samples at a base temperature of ∼275 K (the minimum achievable with our setup), and inducing T-jumps to a final temperature of about 280 K. These specific conditions were chosen to match as closely as possible the temperature used for the equilibrium measurements and previous SM-FRET experiments, while having a sufficiently large T-jump to obtain kinetic traces with good signal-to-noise ratio. T-jump experiments at different final temperatures were performed in a similar way.

### Theoretical Model and Calculations

For all the calculations included in this work we have used a version of the 1D free energy surface model that we have previously developed for the analysis [17], [39] and prediction [41] of protein folding. The model defines a 1D free energy surface as a function of the local order parameter nativeness (*n*), which corresponds to the probability for any given peptide bond in the protein to be in its native conformation (native dihedral angles). Both conformational entropy and stabilization enthalpy scale linearly with protein size (number of aminoacids, *N*) and are defined by the following relationships:
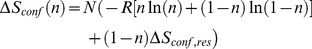
(1)

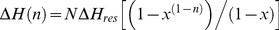
(2)where *ΔH(n)* is a Markov chain, *x* is the characteristic rate for breaking native stabilizing interactions [41] (here set to 0.4 for BBL, which ensures a one-state downhill folding scenario for this protein), *ΔS_conf,res_* is the cost in conformational entropy for fixing a residue in native conformation (here set to 16.75 J·mol^−1^·K^−1^ [17]), *ΔH_res_* is the net gain in stabilization enthalpy per residue (here set to 7 kJ·mol^−1^), and *N* = 40 for BBL. The statistical weight of any species in the unprotonated form defined by a given value of *n* is calculated as
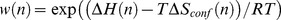
(3)where T is set to the experimental temperature of 279 K. The statistical weights of the species belonging to the protonated form of the protein are calculated as
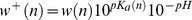
(4)where *pKa(n)* is described here by the simple linear relationship *pKa(n)* = 5+9(*1−n*) (i.e. this definition sets a *pKa* = 5 for fully native BBL, and a *pKa* of ∼11 for a fully unfolded BBL with <n>∼0.3). According to these definitions, the complete partition function is described by
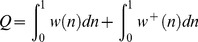
(5)which can also be easily computed for a discretized version of the model as a sum of the statistical weights of the chosen discrete values of n (e.g. every 0.01).

The kinetic description of the model has two components. The first term deals with the kinetics of motion on the free energy surface of the unprotonated and protonated forms, which is taken as diffusive following exactly the same treatment described before [17]. That is, the time-dependent changes in *n* for both protonation forms are calculated using the Szabo matrix formalism for 1D diffusion [54] and setting *D = *650 *n* ^2^·s^−1^. The second term includes the kinetic exchange between the unprotonated and protonated forms of the protein, which we constrain to only occur between species of equal *n*. The overall protonation (on)-rate for each value of nativeness is defined as the sum of three second order rates to account for the acid, base, and amino-transfer catalyzed processes [55] using the following expression:
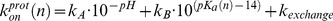
(6)

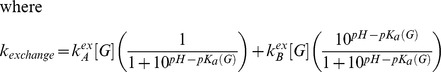

*k_A_* is the rate for acid catalysis (here set to 9·10^9^ M^−1^·s^−1^), *k_B_* is the rate for base catalysis (set to 6·10^6^ M^−1^s^−1^), [G] is the concentration of guanidinium in the experiment, and *k_ex,A_* and *k_ex,B_* are the amino transfer proton exchange rates for the protonated and unprotonated forms of the amine in solution, respectively (here set to 8·10^2^ M^−1^·s^−1^ and 3·10^2^ M^−1^·s^−1^). The deprotonation (off)-rate for each value of nativeness is directly determined by detailed balance:



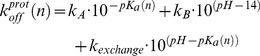
(6)We simulated the effect of chemical denaturants at each pH condition by titrating in small intervals the *ΔH_res_* from 7 kJ·mol^−1^ down to a value of 4 kJ·mol^−1^, which was sufficient to achieve complete unfolding of BBL at all pH conditions. The denaturation midpoint was calculated as the maximum in the derivative of the unfolding curve [56] defined by the changes in average nativeness as a function of denaturing stress. Laser T-jump experiments at the chemical midpoint were simulated by integrating the rate matrix at the denaturation midpoint for each pH condition using as initial populations for all the species those obtained at 275 K (i.e. by setting *T* = 275 in equation 3). The time-dependent probabilities for all the species in the discretized version of the model were then compounded onto the time decay of the overall nativeness signal by calculating:
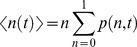
(7)


Single-molecule stochastic kinetic simulations with the model were performed as described before [32], but defining three possible time-dependent transitions from each microstate (defined in terms of nativeness and protonation status): forward, backwards, protonation-deprotonation, according to the following rules:
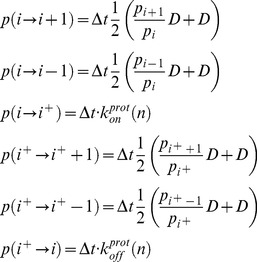
(8)where *D* is the intramolecular diffusion coefficient and Δ*t* is chosen small enough to guarantee that the total probability of jumping from any given microstate (the sum of the three transition probabilities) is always <0.1.
